# High prevalence of refractive errors in an elderly population; a public health issue

**DOI:** 10.1186/s12886-023-02791-x

**Published:** 2023-01-27

**Authors:** Alireza Hashemi, Mehdi Khabazkhoob, Hassan Hashemi

**Affiliations:** 1 Noor Ophthalmology Research Center, Noor Eye Hospital, Tehran, Iran; 2 Department of Basic Sciences, School of Nursing and Midwifery, Shahid Beheshti University of Medical Sciences, Tehran, Iran; 3 Noor Research Center for Ophthalmic Epidemiology, Noor Eye Hospital, Tehran, Iran

**Keywords:** Refractive errors, Myopia, Hyperopia, Elderly, Cross-sectional study

## Abstract

**Purpose:**

To determine the prevalence of myopia and hyperopia and their associated demographic and ocular factors in people 60 years of age and above.

**Methods:**

The sampling was performed using a multi-stage stratified random cluster sampling method. The complete demographic and case history information were collected through an interview. Then, all participants underwent optometric examinations including measurement of uncorrected and best-corrected visual acuity, objective, and subjective refraction. Myopia and hyperopia were defined as a spherical equivalent (SE) refraction worse than -0.50 diopters (D) and + 0.50 D, respectively.

**Results:**

Three thousand three hundred ten of 3791 invitees participated, and the data of 3263 individuals were analyzed for this report. The mean age of participants was 68.25 ± 6.53 (60 to 97) years, and 1895 (58.1%) of them were female (number of male/female participants = 1368/1895). The prevalence of myopia and hyperopia was 31.65% (95% CI: 29.68 -33.61) and 45.36% (95% CI: 43.36 -47.37), respectively. The prevalence of severe myopia and hyperopia was 1.14% (95% CI: 0.73 -1.55) and 2.27% (95% CI: 1.57 -2.97), respectively. Based on the results of multiple logistic regression, the prevalence of myopia had a statistically significant direct relationship with age (OR: 1.04; *p* < 0.001), history of glaucoma surgery (OR:2.75; *p* < 0.001), pseudophakia (OR: 2.27; *p* < 0.001), axial length (OR:3.05; *p* < 0.001), and mean keratometry (OR:1.61; *p* < 0.001). The education level was significantly inversely related to the myopia prevalence. Moreover, a history of glaucoma surgery (OR:0.44; *p* < 0.001), pseudophakia (OR = 0.15; *p* < 0.001), axial length (OR:35; *p* < 0.001) and mean keratometry (OR:0.62; *p* < 0.001) were significantly inversely related to the prevalence of hyperopia. 19% and 40.02% of myopic and hyperopic patients had complete visual acuity after correction of refractive error, respectively.

**Conclusion:**

The prevalence of refractive errors was high in the Iranian elderly population. A large percentage of the elderly still did not have complete visual acuity after the correction of refractive errors indicating the necessity for attention to other ocular diseases in this age group. The history of cataract and glaucoma surgery could be associated with a myopic shift of refractive error.

## Introduction

According to recent reports in 2020, there are an estimated 43.3 million people with blindness and an additional 295 million people with moderate and severe visual impairment worldwide. These figures are expected to increase to 61 million and 474 million in 2050, respectively [1]. Regardless of the severity and type of visual impairment, the World Health Organization (WHO) reports that there are 2.2 billion individuals with visual impairment in the world. Globally, uncorrected refractive errors and cataracts are the leading causes of visual impairment. Uncorrected refractive errors (as the major cause) account for 86.1 million cases of moderate and severe visual impairment [1]. Even cataract cases can be viewed as refractive errors; some of these cases develop residual refractive errors after surgery requiring optical correction, although may not be associated with severe visual impairment [2]. These problems are more pronounced above 50 years of age and impose a significant economic burden on governments [3].

In recent years, various studies have investigated the prevalence of refractive errors in older adults [4–23]. Accordingly, the prevalence of refractive errors in this age group has been reported up to more than 80% [4–23]. Due to the stabilization of ocular growth and slight biometric changes in older ages, the risk factors of refractive error in this age group are expected to be different from adolescents and individuals under 40 years of age. Age-related structural changes in ocular media especially crystalline lens and ocular pathologies are the main causes of refractive errors in this age group [24, 25]. Although a review of the literature indicates various studies in this regard, most previous studies investigated refractive errors in people above 40 years of age and included a small sample of the elderly [3, 5, 6, 8, 9, 13, 14, 16–22, 24–32]. This is while controlling infectious diseases and increasing life expectancy in recent years has led to the increased growth of the elderly population as well as an increase in the proportion of this age group in the total population [33]. A major difference between the elderly population and middle-aged populations is the higher prevalence of various ocular pathologies as well as the history of cataract surgery, which can affect the distribution and pattern of refractive errors. Overall, refractive errors should be considered a health concern in the geriatric population as the major cause of visual impairment associated with decreased quality of life [34, 35]. There have been numerous reports of refractive errors in Iran in recent years [8, 36–39]. However, most of these studies had limitations, including a lack of attention to various influential variables and a small sample size of the elderly. On the other hand, the proportion of the elderly population of Iran, like most countries in the world, has increased compared to previous years. The present population-based study aimed to determine the prevalence of refractive errors and their relationship with demographic and some ocular factors in an Iranian elderly population.

## Methods

The present report is a part of the Tehran Geriatric Eye Study (TGES); a population-based cross-sectional study conducted from Jan 2019 to Jan 2020 in Tehran, the capital of Iran. The sampling frame of TGES was the urban population of Tehran 60 years and older.

Considering a prevalence of 5.2% for visual impairment as the main outcome of the TGES, precision of 1%, and confidence interval (CI) of 95%, the sample size was estimated at 1894 individuals. After applying a design effect of 1.5 and a non-response rate of 10%, the sample size was calculated at 3155 participants, which was rounded up to 3200.

The sampling was performed using a multi-stage stratified random cluster sampling method. First, each of the 22 municipality districts of Tehran was considered as strata and the population 60 years and older in each district was obtained from the National Statistics Center. The population to be selected from each district was determined proportionally to size. Then, a blocked map of each district was prepared and each block was considered as a cluster. A total of 160 clusters with a size of 20 individuals were randomly selected from all 22 districts of Tehran. The number of clusters in each district was proportional to the population of that district. After identifying the selected blocks, a sampling team was sent to the address and located on the southwest side of the selected block, the first house was selected as the head of the cluster. Then, by moving counterclockwise while selecting the next households, all people 60 years and older were invited to participate in the study after explaining the objectives and steps of the study and ensuring the confidentiality of information. If a person wished to participate in the study, informed consent was obtained and an identification card was issued. This process continued until the sample size in each cluster was completed. If there were more than one eligible person in the last household of a cluster, this cluster could include more than 20 individuals. If a household was not present at home, the sampling team would return at another time (preferably in the evening). The study participants were transferred to the examination site on a pre-determined day free of charge. After the participants arrived at the study site, first the complete demographic and case history information was obtained through a face-to-face interview. Then, ocular examinations and ocular biometric measurements were performed.

### Examinations

All optometric examinations were performed by two experienced optometrists in a room with standard illumination. The two examiners showed a high agreement in measuring uncorrected visual acuity (ICC: 0.994) and the spherical equivalent (SE) of subjective refraction (ICC: 0.967) in a pilot of 30 individuals. First, uncorrected distance visual acuity (UCVA) was measured using a LED visual acuity chart (Smart LC 13, Medizs Inc., Korea) at 6 m (m). Then, non-cycloplegic auto-refraction was performed by an auto-refractometer/keratometer (ARK-510A, Nidek Co. LTD, Aichi, Japan). The subjective refraction was performed to determine the optimal distance optical correction and the best-corrected distance visual acuity (BCVA) was recorded. In the next step, all study participants underwent anterior and posterior segment ocular health examination using slit-lamp biomicroscopy)Slit-lamp B900, Haag-Streit AG, Bern, Switzerland) by an ophthalmologist under the dilated pupil conditions (using tropicamide 1% drops). The posterior segment examination was undertaken using a + 90 lens. The ocular biometric measurements were performed using IOL Master 500 (Carl Zeiss Meditec, Jena, Germany).

### Definitions

In line with previous studies [4–9, 11, 13–23, 26, 28–32, 36, 40–44], the SE of auto-refraction was used to define myopia and hyperopia. Myopia and hyperopia were defined as a SE worse than -0.50 diopters (D) and + 0.50 D, respectively. A SE of -0.50 D to + 0.50 D was considered as emmetropia. A person was considered myopic or hyperopic to have at least one of these refractive errors in one eye. To determine the severity of refractive errors, a SE of -0.50 to -3.00 D was considered as mild myopia, -3.00 to -6.00 D as moderate myopia, and above -6.00 D as severe myopia. A SE of + 0.50 to + 2.00 D was defined as mild hyperopia, + 2.00 to + 4.00 D as moderate hyperopia, and above + 4.00 D as severe hyperopia. The cataracts were diagnosed and graded according to the WHO grading system [45]. In this grading system, the severity of crystalline lens opacities is classified into three groups of nuclear, cortical, and posterior subcapsular (PSC) types according to photographic standards. Each type is graded from 0 to 3. Each type of cataract was defined based on the presence of a significant (WHO grade of ≥ 2) nuclear, cortical, or PSC opacity in at least one eye. The participant's pseudophakic status was determined using slit-lamp examination by the ophthalmologist.

### Statistical analysis

In this study, the prevalence of myopia and hyperopia was reported as a percentage with 95% CI. The cluster sampling method was considered in calculating the standard error, and the results were weighted based on the age and sex distribution of the population of Tehran in 2019. The simple and multiple logistic regression models were used to investigate relationships and the odds ratios (OR) with 95% CIs were reported. A *P* value < 0.05 was considered statistically significant.

### Ethical issues

Informed consent was obtained from all participants. For illiterate participants, the goals and steps of the study were fully explained and verbal consent was taken; they also confirmed the consent form with a thumbprint. The principles of the Helsinki Declaration were followed in all stages of this study. The protocol of the study was approved by the Ethics Committee of the National Institute for Medical Research Development (NIMAD) under the auspices of the Iranian Ministry of Health. (grant code: 963,660).

## Results

Three thousand three hundred ten of 3791 invitees participated in the TGES (response rate: 87.3%). The mean age of the participants and non-participants was 68.25 ± 6.55 years and 68.69 ± 7.10 years, respectively. 14.4% and 12.4% of invited men and women did not participate in the study, respectively.

After applying the exclusion criteria, the data of 3263 individuals were analyzed for this report. The mean age of participants was 68.25 ± 6.53 (60 to 97) years, and 1895 (58.1%) of them were female (number of male/female participants = 1368/1895 = 0.72).

**Fig. 1:**
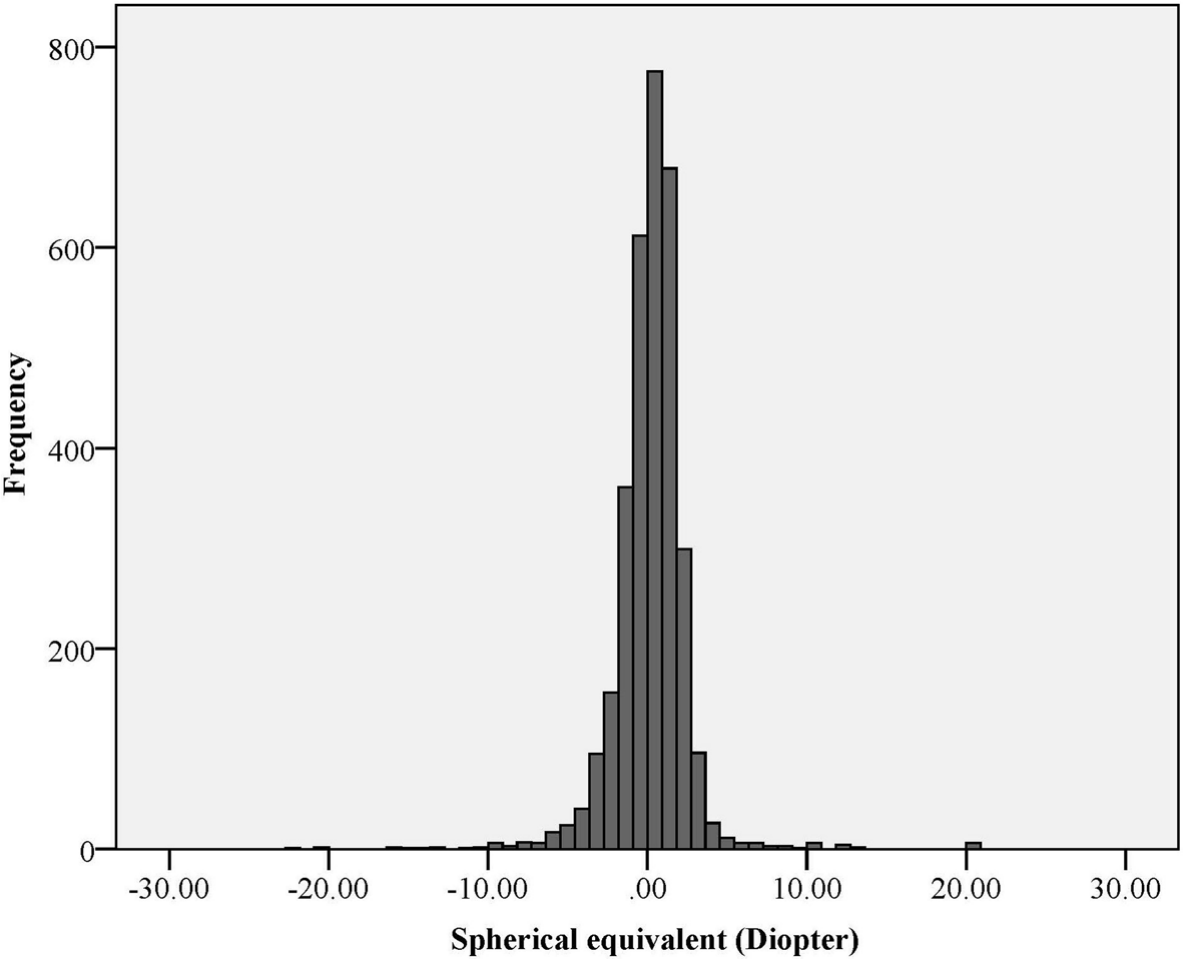
The distribution of spherical equivalent in people 60 years of age and above

**Fig. 2:**
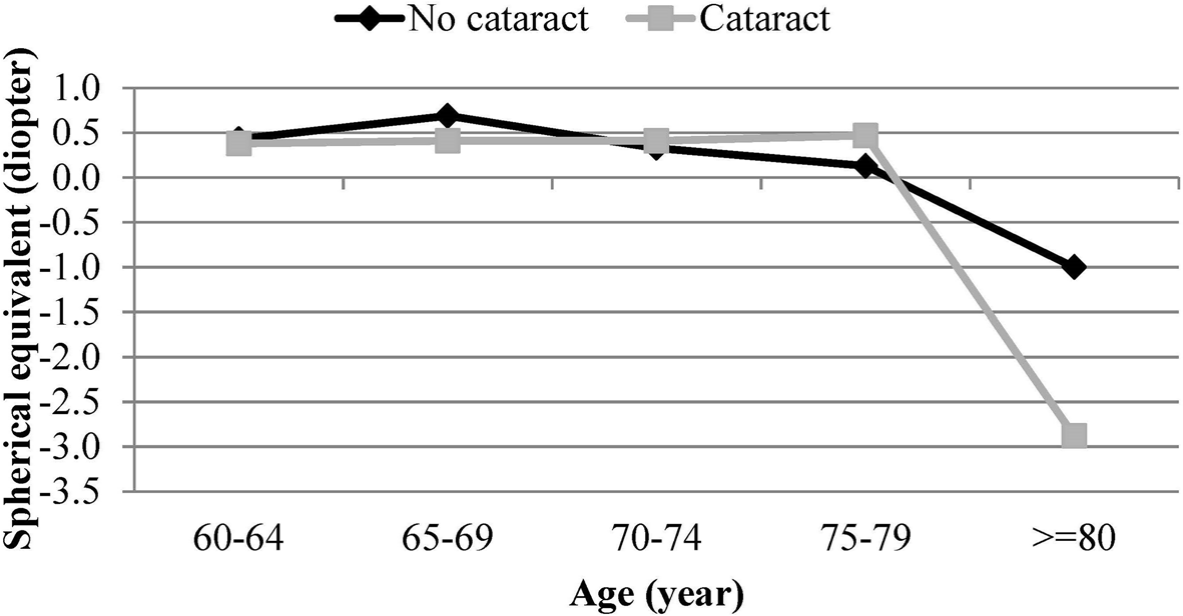
The distribution of spherical equivalent by age and cataract

Figure 1 shows the distribution of SE in the present study. The mean SE was 0.18 ± 2.44 D in the whole sample. The mean SE was 0.13 ± 2.31 D in males and 0.22 ± 2.53 D in females; there was no statistically significant difference in SE between the two sexes. The SE changes in individuals with and without nuclear cataract are shown in Fig. 2. As seen, changes in the SE in the two groups were almost similar up to 80 years. However, after 80 years of age, the myopic shift was significantly higher in individuals with cataract (*P* < 0.001). Table 1 shows the prevalence of myopia, hyperopia, and emmetropia by age, sex, education level, diabetes, history of glaucoma and retinal surgery, and pseudophakia. The total prevalence of myopia was 31.65% (95% CI: 29.68 -33.61) in the present study. There was no statistically significant relationship between sex and the prevalence of myopia after adjusting for age (*P* = 0.579). However, the prevalence of myopia increased from 22.74% (95% CI: 20.21–25.28) in the age group 60–64 years to 45.58% (95% CI: 38.38 -52.78) in the age group ≥ 80 years. Logistic regression showed that each year of advancing age increased the odds of myopia by 1.05 times after adjusting for sex (*P* < 0.001). The odds of myopia in individuals with a history of glaucoma surgery was 1.78 times higher than individuals without a history of glaucoma surgery (*P* = 0.033). However, the history of retinal surgery had no statistically significant relationship with the prevalence of myopia (*P* = 0.353). As seen in Table 1, the prevalence of myopia was 30.39% (95% CI: 26.93 -33.84) and 32.17% (95% CI: 29.85 -34.48) in individuals with and without diabetes, respectively (*P* = 0.390). The odds of myopia in participants with nuclear cataract was 1.28 times higher than individuals without nuclear cataract (*P* = 0.088). As Table 1 shows, the highest prevalence of myopia was related to illiterate people and a decreasing trend in the prevalence of myopia was observed with increasing education level (*P* < 0.001). The prevalence of myopia was 48.13% (95% CI: 43.84 -52.42) and 25.41% (95% CI 23.46 -27.37) in pseudophakic and phakic individuals, respectively (*P* < 0.001). Table 2 shows the severity of myopia in all study participants and by sex. As seen in Table 2, the total prevalence of severe myopia (> 6.00 D) was 1.14% (95% CI: 0.73 -1.55). The mean SE was -10.35 ± 4.21 D in individuals with severe myopia. Table 3 shows the relationship between myopia prevalence and the studied variables in simple and multiple logistic regression models. According to the results of the multiple logistic regression, increasing age, lower education level, history of glaucoma surgery, pseudophakia, longer axial length, and steeper mean keratometry were significantly associated with the increased prevalence of myopia.

**Table 1:** The prevalence of myopia and hyperopia according studied variables

		n	Myopia	Hyperopia	Ammetropia
	Total	3263	% (95%CI)	% (95%CI)	% (95%CI)
Independent variables			31.65(29.68 -33.61)	45.36(43.36 -47.37)	77.01(75.43 -78.59)
Sex	Male	1368	31.22(28.73 -33.71)	45.58(42.67 -48.49)	76.80(74.49 -79.11)
	Female	1895	32.06(29.06 -35.06)	45.15(42.47 -47.83)	77.22(75.01 -79.42)
Age	60–64	1151	22.74(20.21 -25.28)	52.88(49.59 -56.17)	75.62(72.96 -78.29)
	65–69	937	27.99(24.75 -31.23)	49.80(46.29 -53.31)	77.79(74.91 -80.67)
	70–74	627	35.28(31.36 -39.20)	40.71(36.83 -44.59)	75.99(72.45 -79.52)
	75–79	310	44.42(38.55 -50.29)	37.68(32.14 -43.23)	82.10(77.01 -87.18)
	> = 80	238	45.58(38.38 -52.78)	30.24(23.63 -36.85)	75.82(69.10 -82.54)
Glaucoma surgery	No	3191	31.41(29.43 -33.39)	45.58(43.55 -47.61)	76.99(75.38 -78.60)
	Yes	68	44.91(31.94 -57.87)	33.59(21.46 -45.71)	78.49(68.48 -88.50)
Retinal surgery	No	3131	31.44(29.36 -33.51)	45.52(43.46 -47.59)	76.96(75.32 -78.60)
	Yes	130	36.23(26.29 -46.16)	41.75(29.51 -53.98)	77.97(69.29 -86.66)
Diabetes	No	2294	32.17(29.85 -34.48)	45.75(43.26 -48.23)	77.91(76.09 -79.74)
	Yes	969	30.39(26.93 -33.84)	44.44(40.95 -47.93)	74.83(71.82 -77.83)
Nuclear cataract	No any cataract	665	21.37(18.06 -24.69)	57.32(52.90 -61.73)	78.69(75.00 -82.37)
	Yes	528	25.84(21.73 -29.94)	55.91(51.00 -60.82)	81.75(78.45 -85.04)
Education	Illiterate	439	39.32(34.10 -44.55)	40.72(34.82 -46.62)	80.04(75.60 -84.47)
	Primary school	1005	36.66(32.97 -40.35)	41.37(37.66 -45.08)	78.03(75.27 -80.79)
	Guidance School	607	27.69(23.96 -31.42)	45.44(41.33 -49.55)	73.13(69.46 -76.81)
	High school	810	26.19(23.03 -29.35)	51.21(47.25 -55.17)	77.4(73.91 -80.89)
	College	402	26.56(21.43 -31.68)	49.18(42.92 -55.45)	75.74(70.52 -80.97)
Cataract surgery (pseudophakic)	No	2472	25.41(23.46 -27.37)	55.04(52.79 -57.29)	80.45(78.79 -82.12)
	Yes	791	48.13(43.84 -52.42)	19.77(16.4 -23.15)	67.9(64.16 -71.64)

**Table 2:** The prevalence of severity of refractive errors according sex and its mean of spherical equivalent

	**prevalence**			**spherical equivalent(diopter)**		
	Total	Male	Female	Total	Male	Female
Spherical equivalent(diopter)	%(95%CI)	%(95%CI)	%(95%CI)	Mean(95%CI)	Mean(95%CI)	Mean(95%CI)
< -6	1.14(0.73 -1.55)	1.37(0.73 -2.01)	0.91(0.42 -1.41)	-10.08 ± 4.15	-10.37 ± 4.55	-9.65 ± 3.06
3- to -6	4.21(3.42 -5.00)	4.99(3.74 -6.24)	3.45(2.46 -4.43)	-4.14 ± 0.79	-4.04 ± 0.71	-4.29 ± 0.89
-0.5 to -3	26.30(24.51 -28.08)	24.86(22.58 -27.13)	27.7(25.03 -30.38)	-1.39 ± 0.64	-1.38 ± 0.57	-1.40 ± 0.70
-0.5 to 0.5	22.99(21.41 -24.57)	23.20(20.89 -25.51)	22.78(20.58 -24.99)	0.02 ± 0.37	0.03 ± 0.34	0.01 ± 0.39
0.5 to 2	32.77(30.96 -34.58)	34.01(31.33 -36.69)	31.56(29.13 -33.99)	1.15 ± 0.38	1.15 ± 0.35	1.16 ± 0.40
2 to 4	10.32(9.22 -11.41)	9.42(7.88 -10.96)	11.20(9.67 -12.73)	2.52 ± 0.51	2.50 ± 0.47	2.54 ± 0.55
> 4	2.27(1.57 -2.97)	2.15(1.17 -3.13)	2.39(1.36 -3.42)	8.24 ± 4.32	8.44 ± 4.04	8.06 ± 4.55

**Table 3:** Association of myopia and hyperopia with some variables based simple and on multiple logistic regression

		**Simple**		**Multiple**	
		OR(95%CI)	*p*-value	OR(95%CI)	*p*-value
**Myopia**	Age	1.05(1.04 -1.07)	< 0.001	1.04(1.02 -1.05)	< 0.001
	Sex	1.04(0.87 -1.24)	0.667	NS	
	Glaucoma surgery	1.78(1.05 -3.02)	0.033	2.75(1.5 -5.06)	0.001
	Retina surgery	1.24(0.79 -1.95)	0.353	NS	
	Diabetes	0.92(0.76 -1.11)	0.390	NS	
	Nuclear cataract	1.28(0.96 -1.71)	0.088	NS	
	Cataract surgery (pseudophakic)	2.72(2.24 -3.31)	< 0.001	2.27(1.82 -2.83)	< 0.001
	Axial length(mm)	1.54(1.37 -1.73)	< 0.001	3.05(2.38 -3.91)	< 0.001
	Mean keratometry (Diopter)	1.16(1.09 -1.22)	< 0.001	1.61(1.45 -1.79)	< 0.001
	Education				
	Illiterate	1		NS	
	Primary school	0.89(0.69 -1.15)	0.377	0.94(0.69 -1.27)	0.669
	Guidance School	0.59(0.44 -0.79)	< 0.001	0.65(0.46 -0.92)	0.017
	High school	0.55(0.42 -0.72)	< 0.001	0.65(0.47 -0.91)	0.013
	College	0.56(0.4 -0.78)	0.001	0.6(0.4 -0.9)	0.014
**Hyperopia**	Age	0.96(0.95 -0.97)	< 0.001	NS	
	Sex	0.98(0.84 -1.15)	0.828	NS	
	Glaucoma surgery	0.60(0.35 -1.04)	0.071	0.44(0.23 -0.85)	0.015
	Retina surgery	0.86(0.51 -1.44)	0.558	NS	
	Diabetes	0.95(0.8 -1.13)	0.556	NS	
	Nuclear cataract	0.94(0.72 -1.23)	0.671	NS	
	Cataract surgery (pseudophakic)	0.20(0.16 -0.25)	< 0.001	0.15(0.11 -0.19)	< 0.001
	Axial length(mm)	0.69(0.62 -0.77)	< 0.001	0.35(0.27 -0.44)	< 0.001
	Mean keratometry (Diopter)	0.88(0.83 -0.92)	< 0.001	0.62(0.56 -0.69)	< 0.001
	Education				
	Illiterate	1		NS	
	Primary school	1.03(0.79 -1.33)	0.836	NS	
	Guidance School	1.21(0.89 -1.65)	0.214	NS	
	High school	1.53(1.14 -2.05)	0.005	NS	
	College	1.41(0.99 -2.01)	0.059	NS	

*OR* Odds ratio, *CI* Confidence interval, *NS* Not significant

The total prevalence of hyperopia was 45.36% (95% CI: 43.36–47.37) in the present study. The prevalence of hyperopia had no statistically significant relationship with sex (*P* = 0.754). However, the hyperopia prevalence significantly decreased from 52.88% (95% CI: 49.59 -56.17) in the age group 60–64 years to 30.24% (95% CI: 23.63 -36.85) in the age group ≥ 80 years (*P* < 0.001). The prevalence of hyperopia was lower in individuals with a history of glaucoma surgery (*P* = 0.071). There was no statistically significant difference in the prevalence of hyperopia between individuals with a history of retinal surgery and those without a history of retinal surgery (*P* = 0.558). Also, there was no statistically significant difference in the hyperopia prevalence between participants with and without nuclear cataract (*P* = 0.556). The prevalence of hyperopia significantly increased with increasing education level and its lowest prevalence was observed in pseudophakic individuals (*P* < 0.001). 2.27% (95% CI: 1.57–2.97) of the study participants had severe hyperopia (> + 4.00 D), with a mean SE of 8.24 ± 4.32 D. The relationship between the hyperopia prevalence and the study variables was investigated in a multiple logistic regression model, the results of which are shown in Table 3. Based on the results of the multiple regression model, a history of glaucoma surgery, pseudophakia, axial length, and mean keratometry (in diopters) were significantly inversely related to the prevalence of hyperopia.

The UCVA of myopes and hyperopes increased by 0.28 and 0.27 decimal on average with optimal correction (BCVA), respectively. 19% of myopia patients had complete visual acuity (20/20) after correction of refractive error, this finding was observed in 40.02% of hyperopic patients. Presenting visual acuity (PVA) was 0.16 and 0.12 decimal on average better compared to UCVA in myopes and hyperopes, respectively.

## Discussion

Although various studies have investigated the prevalence of refractive errors in people above 40 years of age worldwide [3, 5, 6, 8, 9, 13, 14, 16–22, 24–32], the present population-based study specifically examined refractive errors in an elderly population aged 60 years and above using a large sample size. Non-exclusion of individuals with a history of ocular surgery is one of the important points that should be considered first. The high prevalence of ocular surgeries in older ages would cause a significant number of subjects to be excluded from the study. It should be noted that after the age of 60 years, a large number of people have experienced at least one ocular surgery or ophthalmic intervention. Anyway, these people have refractive errors regardless of their surgical history, and most of them need optical correction. So they are a special group that should be considered in terms of refractive error burden and public health issues.

According to the results, 31.65% of the participants in the present study were myopic. A summary of the findings of previous similar studies is shown in Table 4.

**Table 4:** Summary of other studies concerning myopia and hyperopia in the middle-aged and elderly

First author	Location	Age	Myopia(SE < -0.5)	Hyperopia(SE > 0.5)
Pan	China (Yunnan) [27]- Yi Ethnicity	≥ 50	8.1	-
Pan	China (Yunnan) [27]- Han Ethnicity	≥ 50	10.3	-
Ezelum	Nigeria (Anambra State) [6]	≥ 40	14.1	51.1
Attebo	Australia (Blue Mountains) [4]	49–97	15.0	57
Wang	China (Gansu) [16]	40–80	16.4	26.2
Wensor	Australia (Victoria) [40]	≥ 40	17.0	18
Wickremasinghe	Mongolia [31]	≥ 40	17.2	32.9
Cheng	Taiwan [5]	≥ 65	19.4	59
Liang	China [9]	40–79	19.4	1.6
Yoo	Korea (Namil) [22]	≥ 40	20.5	41.8
Xu	China (Eastern) [20]	≥ 60	21.1	-
Wu	USA (Barbados) [19]	40–84	21.9	46.9
Xu	China [21]	40–90	22.9	20
Hashemi	Iran (Shahyoun & Kajour) [8]	≥ 40	23.6	34.1
Wang	USA(Beaver Dam) [15]	43 to 84	26.2	49
Wang	China (Yunnan) [29]	40–80	26.35	19.89
Raju	India [12]	≥ 40	27.0	18.7
Pan	Singapore (Indian)- (SINDI) [10]	> 40	28	35.9
Wang	China (Qinghai) [30]	50–79	28.56	22.82
Wang	China (Inner Mongolia) [14]	40–80	29.4	28.4
Nakamura	Japan (Kumejima) [32]	≥ 40	29.5	34.1
Saw	Singapore [41]	40–80	30.7	27.4
Hashemi	Iran (Khaf) [36]	> 40	32.5	27.9
Krishnaiah	India [26]	> 40	34.6	18.4
Kim	Korea [42]	> 40	34.7	34.8
Wong	Singapore [18]	40–79	35.0	35.9
Varma	USA (Chinese American) [28]	≥ 50	35.1	40.2
Wolfram	Germany (Gutenberg) [17]	35–74	35.1	31.8
Rim	Korea [43]	≥ 40	35.3	24.9
Ziaei	Iran (Yazd) [23]	40–80	36.5	20.6
Pan	Singapore [11]	> 40	38.9	31.5
Sawada	Japan [13]	≥ 40	41.8	8.2
Ueda	Japan (Hisayama) [44]	≥ 40	45.8	-
Gupta	Myanmar [7]	≥ 40	51.0	15

*SE* Spherical equivalent

The number of studies reporting the prevalence of myopia in the elderly is much higher than the studies listed in Table 4. However, due to significant differences in the definition of myopia and age distribution, we only included studies that could provide more accurate comparisons. Globally, the prevalence of myopia in individuals above 40 years of age has been reported in a wide range from 8% in China [27] to 51% in Myanmar [7]. It should be noted that about half of the elderly over the age of 80 years in the present study had myopia. According to a previously published review article, the highest and lowest prevalence of myopia in the elderly worldwide were related to Southeast Asian and African countries, respectively [46]. The relatively high prevalence of myopia in the present study may be due to the non-exclusion of pseudophakic individuals since according to the results of this study, the prevalence of myopia in pseudophakic patients is significantly higher. However, due to the relationship of nuclear cataract with myopia, these people may also be myopic if they did not undergo cataract surgery.

According to the results of the present study, 45.36% of the participants had hyperopia. The prevalence range of hyperopia is wider than myopia in people above 40 years of age worldwide. As seen in Table 4, the prevalence of hyperopia in this age group has been reported from 1.6% in China to over 50% in countries such as Nigeria, USA, and Iran.

According to a review article that included more than 150 papers, [46] the highest and lowest prevalence of hyperopia in the elderly were seen in African (38.6%) and European (23%) countries, respectively. The results of the present study indicate a high prevalence of hyperopia in the Iranian elderly population compared to previous studies, although the prevalence of hyperopia decreased at older ages. Previous studies from Iran also showed that hyperopia in Iran was more common than myopia in adults and children [8, 24, 37–39]. The association of this refractive error with presbyopia can create difficult vision conditions for the elderly that should be taken into account in the Iranian population. As seen, there are significant differences between the results of various studies, especially regarding the prevalence of hyperopia. Various factors can be proposed for these discrepancies, including lifestyle, genetic, racial, and ocular biometric differences. A noteworthy point in the findings of the present study is that 12.5% and 5.3% of the participants had hyperopia worse than 2 D and myopia worse than 3 D, respectively. Certainly, this finding could not be due to cataract surgery, as the error in calculating the intraocular lens power is mostly in the range of 1 D [2]. This prevalence finding is relatively high compared to other studies, [37, 39] and these are cases that may experience visual impairment if not corrected. It should also be noted that a large percentage of these cases, in addition to the significant refractive error, may have other serious problems such as retinal diseases.

Evaluating the factors affecting myopia and hyperopia in this age group is more sophisticated than other age groups. Most of the elderly have ocular pathological problems or a history of ocular surgery due to their old age, which can affect refractive errors. As the results showed, about 77% of the participants in the present study were myopic or hyperopic regardless of astigmatism, indicating the high prevalence of these two refractive errors in presbyopic ages and the need for attention. Older age, a history of glaucoma surgery, and pseudophakia (history of cataract surgery) were risk factors of myopia, and a history of glaucoma surgery and pseudophakia were the factors associated with hyperopia in the present study. It should be noted that the factors associated with myopia were mostly inversely related to hyperopia. So in this section, the factors that were found as a risk factor for myopia (and the protective factor of hyperopia) are discussed.

Regarding myopia changes with age, this finding was found in the multiple logistic regression model in the presence of nuclear cataract, while nuclear cataract did not remain as an independent risk factor of myopia in the final model. This finding contradicts many previous studies, but it should be noted that most of the previous studies examined people above 40 years of age [5, 13, 32, 38]. It seems that due to the presence of mild degrees of other types of cataracts in this age group and the association of hyperopia with some of them; no statistically significant relationship was observed between myopia and nuclear cataract in the present study. According to the definition criteria of cataract in the present study, some individuals were not considered as nuclear cataract cases because they had less grade 2 opacity. These cases may have mild degrees of other cataract types. The other ocular pathologies in the elderly such as glaucoma may also contribute to the relationship between myopia prevalence and age. A history of glaucoma surgery was found to be a risk factor of myopia in the present study. This finding seems to be related to the refractive condition of glaucoma patients before any intervention or surgery. Previous studies have shown that there is a relationship between glaucoma and myopia, [47] so part of this relationship may be due to myopic refractive error of glaucoma patients before surgery.

The results of the present study showed that the odds of myopia in pseudophakic people was significantly higher than in phakic individuals. This finding is important in terms of public health. The presence of clinically significant refractive errors in pseudophakic individuals is unexpected and can be due to an error in calculating the intraocular lens power prior to cataract surgery or improper incision during surgery [2]. Various factors should be considered in the prevalence of myopia in pseudophakic patients. First, myopic target refraction is a common method used by surgeons today to help patients near vision after surgery [48]. Second, errors in the ocular biometry process and ultimately improper determination of intraocular lens power due to the use of traditional biometric technologies and formulas can be another cause of high prevalence of myopia in pseudophakic individuals [49]. In addition, when these people underwent surgery, the use of toric intraocular lenses to correct astigmatism was not common and the results of the surgery were associated with residual post-operative astigmatism. This residual astigmatism causes a shift of SE toward myopia. On the other hand, other factors such as common ocular comorbidities in this age group and untreated posterior capsular opacification (PCO) causing myopia should not be ignored [50].

In this study, we did not find a significant relationship between sex with myopia and hyperopia, while in some previous studies the prevalence of myopia was reported to be higher in men than women, [51, 52] or no significant difference was found between males and females [4, 53, 54].

The main reason for this finding is probably controlling the effect of important biometric components such as axial length and corneal curvature in the present study. Most previous studies attributed higher prevalence of myopia in men to longer axial length or higher prevalence of cataracts [8, 55]. In the present study, the axial length and the corneal curvature (mean keratometry) were significantly related to the prevalence of myopia (direct relationship) and hyperopia (indirect relationship) and it was shown that by controlling the effect of these components, we should not expect a significant difference between the two sexes in terms of myopia and hyperopia prevalence. Higher prevalence of myopia in individuals with longer axial length and steeper corneal curvature is a finding that has been shown in various studies and the effects of these two factors on refractive errors are well-known [56, 57]. We intend to investigate with more detail the role of biometric components on refractive errors in a separate report, and our main purpose in presenting them in this report was to control their effects on other variables.

One of the most important findings of the present study was the rate of increase in patients corrected visual acuity compared to uncorrected visual acuity in myopic and hyperopic cases. The mean visual acuity improvement was 0.28 and 0.27 decimal on average in myopic and hyperopic cases, respectively. 19% and 40% of myopic and hyperopic patients had complete visual acuity after correction of refractive error, respectively. This finding confirms the associated pathological problems and ocular comorbidities in this age group especially in those with myopia, and suggests that other problems such as retinal and lens pathologies could reduce visual acuity in the elderly. This finding should be given serious attention.

The present report had strengths and limitations. The population-based investigation of refractive errors in a large sample of the elderly population ≥ 60 years by demographic and some ocular variables is the strength of this study. One of the important limitations of this study was the greater participation of women; although, we tried to control this issue by standardizing according to age and sex distribution.

## Data Availability

The datasets used and/or analyzed during the current study available from the corresponding author on reasonable request.
